# Immunogenomic Classification of Colorectal Cancer and Therapeutic Implications

**DOI:** 10.3390/ijms18102229

**Published:** 2017-10-24

**Authors:** Jessica Roelands, Peter J. K. Kuppen, Louis Vermeulen, Cristina Maccalli, Julie Decock, Ena Wang, Francesco M. Marincola, Davide Bedognetti, Wouter Hendrickx

**Affiliations:** 1 Tumor Biology, Immunology, and Therapy Section, Immunology, Inflammation, and Metabolism Department, Division of Translational Medicine, Sidra Medical and Research Center, PO Box 26999 Doha, Qatar; jroelands@sidra.org (J.R.); cmaccalli@sidra.org (C.M.); 2 Department of Surgery, Leiden University Medical Center, 2333 ZA Leiden, The Netherlands; P.J.K.Kuppen@lumc.nl; 3 Academic Medical Center Amsterdam, Center for Experimental Molecular Medicine, 1105AZ Amsterdam, The Netherlands; l.vermeulen@amc.uva.nl; 4 Qatar Biomedical Research Institute, Hamad Bin Khalifa University, Qatar Foundation, PO Box 5825 Doha, Qatar; jdecock@hbku.edu.qa; 5 Office of the Chief Research Officer (CRO), Sidra Medical and Research Center, PO Box 26999 Doha, Qatar; ewang@sidra.org (E.W.); francesco.marincola@abbvie.com (F.M.M.)

**Keywords:** colorectal cancer, tumor microenvironment, immune contexture, immunologic constant of rejection, consensus molecular subtypes, immunotherapy, combination therapy

## Abstract

The immune system has a substantial effect on colorectal cancer (CRC) progression. Additionally, the response to immunotherapeutics and conventional treatment options (e.g., chemotherapy, radiotherapy and targeted therapies) is influenced by the immune system. The molecular characterization of colorectal cancer (CRC) has led to the identification of favorable and unfavorable immunological attributes linked to clinical outcome. With the definition of consensus molecular subtypes (CMSs) based on transcriptomic profiles, multiple characteristics have been proposed to be responsible for the development of the tumor immune microenvironment and corresponding mechanisms of immune escape. In this review, a detailed description of proposed immune phenotypes as well as their interaction with different therapeutic modalities will be provided. Finally, possible strategies to shift the CRC immune phenotype towards a reactive, anti-tumor orientation are proposed per CMS.

## 1. Introduction

Colorectal cancer (CRC) progression is influenced by a complex interplay between cancer cells and the tumor microenvironment (TME). Current clinical stratification methods primarily focus on the tumor cell component. Prognosis and treatment is dependent on the localization of the tumor (either colon or rectum) and Tumor Node Metastasis (TNM) staging. Patients clinical outcomes vary widely within the same TNM-stage, illustrating that this stratification does not provide optimal prognostic information [1]. More recently, microsatellite instability (MSI) testing has been indicated in various clinical settings and provides an additional classification system for CRC [2,3]. MSI is a consequence of deficient DNA mismatch repair mechanisms, leading to an increased mutational and neoantigenic load and has been associated with high levels of tumor-infiltrating lymphocytes (TILs), a good clinical prognosis and decreased risk of metastasis [4]. This classification adds significant prognostic power, however, still does not reflect the full complexity of the tumor and its interactions with the TME.

The impact of immune cells on tumor progression has been extensively reported in various cancer types [5,6,7,8,9,10]. The effect of the tumor immune infiltrate on clinical outcome depends on a multitude of factors, including tumor type, the specific cellular composition of the infiltrate, its functional orientation and intratumoral localization and tumor- and host genetics. Immune cells can on the one hand have a tumor suppressive effect and control tumor growth during the immune surveillance and equilibrium phases, as defined by the immunoediting theory [11]. These phases result in either the complete elimination of the cancerous cells or eventual tumor evasion of the immune system. On the other hand, tumor progression can be induced by chronic inflammation, characterized by a polarized immune infiltrate that overturns adaptive immune responses stimulates proliferation and survival of cancerous cells and promotes angiogenesis and metastasis [12]. In the last decade, immune-based classifications showed promise of providing new prognostic and predictive tools for both conventional treatment options, such as chemotherapy [13] and radiotherapy [14], as well as immunotherapy [15].

In this review, we will provide an overview of evidence for factors determining the effect of the tumor immune environment on clinical outcome of cancer patients, with a specific focus on CRC. In addition, immune-based classification strategies for tumors will be evaluated, including their clinical prognostic and predictive values. Finally, an overview of potential immunotherapeutic approaches applicable to different molecular subtypes of CRC will be provided.

## 2. Prognostic Signatures

### 2.1. Tumor-Infiltrating Lymphocytes (TILs)

The TME is composed of epithelial cells, blood and lymphatic vessels, stromal cells, and infiltrating immune cells, including T lymphocytes (e.g., effector T lymphocytes (CTLs), T-helper (T_H_) cells, T-regulatory cells (T-reg)), B cells, natural killer (NK) cells, dendritic cells (DCs) cells, macrophages, myeloid derived suppressor cells (MDSC), and granulocytes. The composition of the TME varies substantially between cancer types, and even between patients with a similar cancer type. For CRC, a subset of patients show a high degree of immune cell infiltration [16], whereas others demonstrate a high presence of mesenchymal stromal cells in their TME, e.g., cancer associated fibroblasts (CAFs) [17], both of which demonstrate relevant cross-talk with the tumor.

The context of the TME is essential to determine the effect of infiltrating immune cells on clinical outcome. First, the prognostic value of different types of immune cells is dependent on tumor type, as reviewed by Fridman et al. [5]. Overall, the infiltration of CD8^+^ T cells is associated with improved clinical prognosis in the majority of cancer types [18]. Exceptions are represented by kidney cancers and glioma, where an inverse association between immune infiltration and prognosis has been observed [19]. For CRC, recent studies have attributed a good prognosis to infiltration of Th_1_ cells [20,21], T follicular helper (T-fh) cells, M1 macrophages [22], DCs [23] and NK cells [24], while presence of M2 macrophages, MDSCs [25], Th_17_ [26,27] and B cells [28] has been associated with poor outcome. Second, the intra-tumoral localization of these immune cells has shown to influence prognostic significance [24]. Lymphocyte densities have been compared between different tumor compartments: center of the tumor (CT), invasive margin (IM) and lymphoid aggregates termed tertiary lymphoid structures (TLS). As TLS show structural similarities with lymph nodes, including a T zone with mature DCs, a profound role in shaping of the tumor immune structure has been proposed [29]. High densities of memory CD45RO^+^ T cells and CD8^+^ T cells in both the CT and the IM have been associated with a favorable clinical outcome [24]. Likewise, infiltration of mature DCs, follicular T helper cells, and B cells in TLS correlate with good prognosis [24]. In a mouse model of CRC, intravenous injected immune cells were attracted to TLSs, suggesting a role for T cell recruitment to the tumor [30]. Considering the proposed role of TLS in cancer and the observed clinical impact of defined immune cells when located specifically in TLS, mechanistic insights into TLS functionality and development in CRC could provide a better understanding of the complex interactions in the TME.

An effort to translate these findings to the clinic has been undertaken internationally. The “Immunoscore”, based on the histological quantification and localization of cytotoxic and memory T cells in CT and IM, has been developed and an international consortium has been put in place to evaluate this scoring system in colon cancer [6,31]. The most recent results support the initial evidence: time to recurrence was significantly larger in patients with a high immunoscore [31], and this stratification appears to have additional prognostic value when combined with conventional TNM-staging [32,33]. Moreover, the immunoscore has proven to be a stronger predictor of patient survival compared with MSI status [34].

### 2.2. Gene Signatures

**Figure 1:**
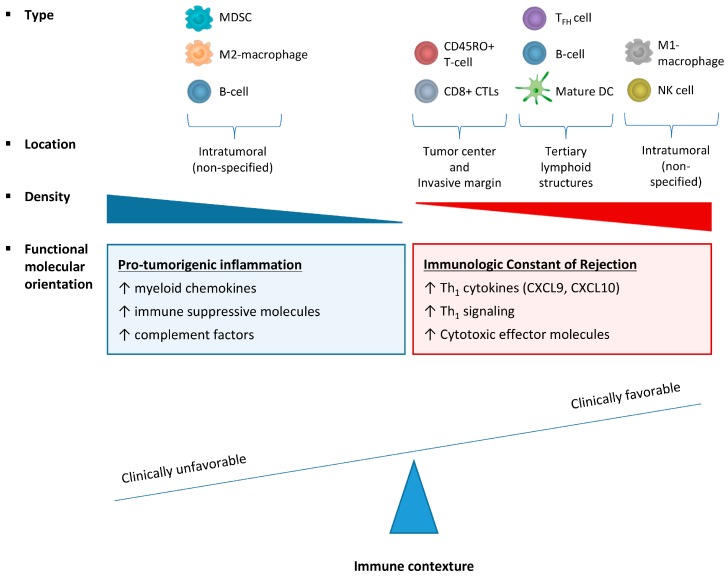
Attributes of an unfavorable versus favorable intratumoral immune contexture. The immune contexture of a tumor is defined by the combination of type, location and density of infiltrating immune cells and functional molecular orientation of the tumor microenvironment. A favorable immune contexture is defined by infiltrating CD45RO^+^ memory T cells, CD8^+^ cytotoxic T-lymphocytes (CTLs) to the tumor center and invasive margin and infiltration of follicular T-helper cells (T_FH_), B-cells and mature dendritic cells (DC) to tertiary lymphoid structures and infiltration of M1-macrophages and NK cells. A favorable functional molecular orientation is characterized by expression of immunologic constant of rejection (ICR) genes, including T-helper type 1 (Th_1_) cytokines (e.g., Chemokine (C-X-C motif) ligand 9 (CXCL9), and -10 (CXCL10)), Th_1_ signaling and cytotoxic effector molecules. An unfavorable immune contexture in terms of patient prognosis is characterized by intratumoral myeloid derived suppressor cells (MDSC), M2-macrophages and B-cells associated with pro-inflammatory gene expression, including myeloid chemokines, immune suppressive molecules and complement factors.

The results of analysis of the abundance and location of TILs demonstrate the impact of the immune infiltrate on cancer progression. However, essential aspects that determine immune response, including the expression of chemokines, cytokines, Major Histocompatibility Complex (MHC) molecules, co-stimulatory, immunomodulatory and cytotoxicity mediating molecules are not encompassed by this analysis. The combination of all relevant functional attributes of the tumor immune infiltrate have been defined as the functional orientation, which in combination with the type, location and density of TILs has been described as immune contexture [5] (Figure 1). Multiple research groups have aimed to characterize the functional orientation of the tumor in relation to patient prognosis. The widespread use of gene expression profiling has enabled the identification of specific immune-related signatures associated with a favorable clinical outcome for cancer patients.

The identification of specific pathway activations consistently associated with tissue-specific destruction in multiple pathological conditions, e.g., graft versus host disease, autoimmunity, pathogen clearance, as well as tumor rejection, has led to the formulation of the immunologic constant of rejection (ICR) [7,35,36,37,38,39]. Characteristics of this phenotype are a Th_1_ immune polarization, upregulation of associated chemokines (Chemokine (C-X-C motif) receptor 3(CXCR3) and C-C chemokine receptor type 5 (CCR5) ligands) and cytotoxic effector molecules. The gene signature associated with this phenotype, additionally including immune regulatory genes *IDO1*, *FOXP3*, *PDCD1*, *CTLA4*, and *CD274/PD-L1*, has been associated with an improved prognosis in multiple cancer types [40,41,42]. Consensus clustering based on this gene signature categorizes tumors based on immune phenotype, enabling prognostication. Although encompassing the immune functional orientation, ICR classification does not include immune cell localization. Additionally, the specific cellular origin of expression profiles cannot directly be derived from the ICR gene expression signature. Information on the relative abundance of specific immune cell subpopulations is therefore not obtained.

A somewhat different strategy to utilize gene expression data of cellular heterogeneous tumor samples to estimate the cellular composition of these bulk tissue samples, is presented by the implementation of bioinformatic methods that rely on transcriptomic data of individual cell populations. Specific gene sets that reflect distinctive transcriptional profiles for immune cell types can be used to calculate scores based on average expression [19,43] or single sample enrichment values of these signatures [24,44]. Around 40 leukocyte subpopulations have been defined with these approaches [24,43,45,46]. Another strategy is cell-type identification by estimating intra-sample relative expression of transcripts such as the one proposed by CIBERSORT, which deconvolutes the relative fractions of 22 leukocyte subtypes using genes differentially (but not uniquely) expressed by isolated human hematopoietic cells [47]. Although this method can determine the cellular composition of the tumor immune infiltrate, the overall abundance of immune cells in the sample is not derived. A recently developed algorithm, the Microenvironment Cell Population-counter (MCP-counter), relies on transcriptomic markers that are uniquely expressed in the cell populations of interest [48]. Such approach could estimate the “absolute” abundance scores for eight immune and two stromal cell populations.

Using single-sample gene set enrichment analysis (ssGSEA), Bindea et al. [24] demonstrated an overrepresentation of genes specific for Th_1_, Tγδ, CTLs, macrophages and mast cells in CRC patients with prolonged disease-free survival, while patients with unfavorable outcome exhibited increased intratumoral expression of genes specific for eosinophils, Th_2_, Th_17_, T-regs and NK cells [24]. A recent study, performed by Chifman et al. [19], has used unsupervised clustering and gene ontology enrichment analysis to identify distinct immune-enriched gene clusters that reflect specific combinations of immune cell subpopulations conserved across multiple cancer types [19]. The immune infiltration was confirmed to be the primary source of the gene expression, as verified by comparison with the gene expression of flow-sorted leukocytes and cancer cell lines. As would be expected, different immune gene clusters were shown to have different prognostic impact dependent on cancer type. In colon cancer, the B/P/T/NK gene cluster, characterized by B cells, plasma B cells, T cells and NK cells, significantly associated with a good patient prognosis, followed by the T/NK gene cluster. Subgroup analysis of tumor stage II patients demonstrated an opposite association with survival for various gene clusters reflecting immune cells of myeloid origin, e.g., B cells, monocytes and DCs [19]. Histological studies have also previously reported inverse correlations with clinical outcome for DCs [49], macrophages [25] and B cells [28]. These findings demonstrate the bi-directional effects of the immune infiltrate can have on cancer progression depending on its specific cellular composition.

### 2.3. Effect of Immune Cell Infiltration Depends on the Tumor Microenvironment (TME)

**Figure 2:**
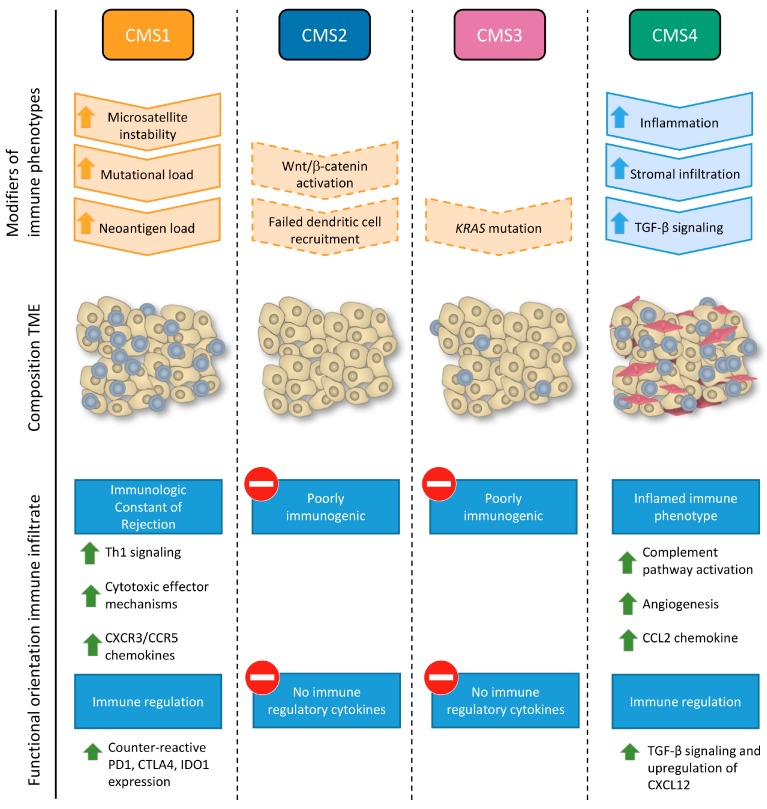
Intratumoral immune phenotypes associate with consensus molecular subtypes (CMS) of colorectal cancer. The transcription- and mutational profiles of consensus molecular subtypes are associated with characteristic intratumoral immune phenotypes. Proposed modifiers of the immune phenotype, either genetic (in orange) or environmental (in blue), supported by experimental evidence in colorectal cancer (solid borders) or supported by evidence in other cancer types (dashed borders) are shown. Both CMS1 and CMS4 tumor microenvironments (TME) are characterized by high levels of TILs (blue), while CMS4 is also infiltrated with cancer-associated fibroblasts (red). CMS1 and CMS4 display divergent functional orientations of their immune infiltrate: while CMS1 tumors display a favorable orientation defined by expression of Immunologic Constant of Rejection (ICR) genes, associated with counter-active upregulation of immune checkpoint molecules; CMS4 tumors have an unfavorable, inflamed immune phenotype, characterized by transforming growth factor beta (TGF-β) signaling, complement activation and increased angiogenesis. CMS2 and CMS3 are both poorly immunogenic characterized by exclusion of TILs from the tumor site and minimal expression of immune-related transcripts. CXCR3/CCR5: Chemokine (C-X-C motif) receptor 3/C-C chemokine receptor type 5, PD1: programmed death protein 1, CTLA4: cytotoxic T-lymphocyte-associated protein 4, IDO1: Indoleamine-pyrrole 2,3-dioxygenase, CCL2: chemokine (C-C motif) ligand 2, CXCL12: Chemokine (C-X-C-motif) ligand 12.

The analysis of the effects of the immune infiltrate in specific subgroups of patients, helps to determine factors that influence the effect immune cells have on clinical outcome of CRC patients. For example, in breast cancer, the prognostic value of an immune-based classification system is dependent on both the proliferation status and molecular subtype of the breast tumor [43]. For CRC, an international consortium of experts has recently introduced a gene expression-based classification system, defined as the “consensus molecular subtypes” (CMSs) of CRC, based on consensus between different previously stratification systems proposed by different research groups [50]. Briefly, four CMSs of CRC are recognized: CMS1 is characterized by MSI, mutations in the *BRAF* oncogene, a diffuse immune infiltrate, composed of Th_1_ cells and CTLs and a strong activation of immune evasion pathways; CMS2 tumors showed high chromosomal instability and activation of *Wnt* and *MYC* pathways; CMS3 displayed frequent *KRAS* mutations and disrupted metabolic pathways and CMS4 is characterized by high expression of mesenchymal genes, stromal infiltration, angiogenesis and transforming growth factor beta (TGF-β) activation (Figure 2). The four subtypes have differential prognosis, with CMS4 tumors displaying worse overall and relapse-free survival [50]. In a recent study, Becht et al. [18], demonstrated that the composition of the TME varies significantly between CMSs. Both CMS1 and CMS4 showed high levels of infiltrating CD8^+^ CTLs and CD68^+^ macrophages, as determined by the MCP-counter methodology. Stromal cell infiltration was significantly higher in CMS4 tumors compared with other CMSs. Gene expression analysis of chemokines, inflammatory molecules, immunoregulatory genes, MHC molecules, complement factors and angiogenesis demonstrated significant differences between CMS1 and CMS4, with CMS1 exhibiting a marked Th_1_ polarization, T cell attracting chemokines, and CMS4 showing high expression of complement components, myeloid chemokine chemokine (C-C motif) ligand 2 (CCL2), angiogenic factors and immunosuppressive molecules [18]. These findings illustrate that evaluation of the functional molecular orientation of the TME provides additional information beyond immune cell infiltration numbers.

A comprehensive analysis of cell-specific gene expression using fluorescence-activated cell sorting (FACS)-sorted primary CRC samples, isolating leukocytes, fibroblasts, endothelial- and epithelial cells, revealed that transcripts associated with poor clinical prognosis are predominantly originating from the tumor-associated stromal cells and endothelial cells [51]. Many characteristics of this poor prognosis subgroup overlap with CMS4 tumors, including their prognosis, high expression of stromal-derived genes and TGF-β signaling. For this reason, it seems that stromal cells determine the fate of these tumors, prevailing over the abundantly infiltrated immune cells. Using the terminology of the previously mentioned breast cancer paper by Miller et al. [43], these tumors would fall into an “immune benefit disabled (IBD)” category. Strikingly, the expression profiles of IBD breast tumors show very similar expression profiles with CMS4 colon tumors. TGF-β was predicted as key transcription regulator of IBD breast tumors [43]. Given the potent immunosuppressive role of TGF-β [52,53,54], it is reasonable to speculate that this cytokine is responsible for the shift in functional orientation of the immune infiltrate in these immunosuppressed cancer subtypes, possibly by a similar mechanism across different cancer types.

As opposed to CMS1 and CMS4 tumors that are characterized by high levels of immune infiltration, although antagonistic regarding their functional orientation, CMS2 and CMS3 are devoid of immune cell infiltration [50]. CMS1 tumors that escape immune surveillance are characterized by upregulation of *PDCD1*, *CTLA4* and *CD274/PD-L1*, while CMS4 tumors exhibit TGF-β signaling and *CXCL12* expression [18]. In contrast, the expression of all these immunosuppressive genes in CMS2 and CMS3 is low, suggesting a different mechanism of immune escape in these subtypes [18,50]. Increasing evidence suggests a role for tumor-intrinsic oncogenic pathways leading to complete immune avoidance by exclusion of T cells from the tumor site [55]. For example, mutation-driven upregulation of Wnt/-catenin correlates with T cell exclusion in melanoma, suggesting failed recruitment of DCs caused by β-catenin-mediated suppression of *CCL4* gene transcription [56]. This immune escape mechanism might also apply to CMS2 colon tumors characterized by activation of the Wnt pathway. In lung tumors with mutated *KRAS*, co-mutation of tumor suppressor gene *LKB1* serine/threonine kinase was inversely correlated with numbers of intratumoral T cells. In a mouse model of KRAS-driven lung cancer, co-mutation of *LKB1/STK11* was shown to promote neutrophil recruitment and proinflammatory cytokine production, suggesting an additional mechanism of immune evasion [57]. As CMS3 colon tumors are characterized by mutated *KRAS* and since *LKB1* mutation also occurs frequently in colon cancer [58], this mechanism could be a relevant immune escape mechanisms in this context as well. Another strategy of CRC tumors to evade the immune system is downregulation of MHC class I, hereby reducing presentation of tumor-associated antigens and reducing recognition by immune cells [59,60]. Upregulation of non-classical human leukocyte antigen-G (*HLA-G*) and *HLA-E* by CRC cells inhibits NK cell function, representing an additional mechanism of immune evasion [60,61,62,63]. Increased expression of *HLA-E* and *HLA-G* genes is primarily observed in CMS1 tumors, but is also found in a subset of CMS2, CMS3 and CMS4 tumors [18]. Since CMS2 and CMS3 tumors account for approximately 50% of colon tumors [50], identification of potential mechanisms of immune evasion is highly relevant. In perspective of the development of immunotherapies, it is important to increase our understanding of immune avoidance to enable the development of strategies to render tumors more immunogenic.

Evidence of immune gene expression as major determinant of clinical outcome is provided by a study that compared gene expression of microsatellite stable (MSS) and MSI CRC tumors [34]. Overall, MSI tumors showed higher expression of immune signatures and improved survival compared with MSS tumors. This is suggested to be caused by the higher mutational load observed in MSI tumors, resulting in a higher number of neo-epitopes per tumor [34]. Within the MSS group, a subset of patients showed increased expression of immune genes, indicating that increased mutational load caused by MSI is not the sole factor determining immune infiltration. This specific MSS subset had prolonged disease free survival. Analysis showed that the risk of relapse was dependent on the immune gene expression, while differentially expressed tumor-related genes had no impact on outcome in this setting. Similarly, patients with MSI tumors, which mostly have similar overall expression profiles, had improved clinical outcome in a subset of patients that exhibited high expression of immune cell markers [34]. These findings illustrate that the immune infiltrate has a direct influence on patient outcome and is not just a bystander product reflecting tumor cell status.

### 2.4. Immune Signatures in CRC Metastasis

Although extensive research has been performed to define the immune phenotype of primary CRC tumors, the characterization of metastasized CRC, which represents the main cause of colon cancer-associated death [64,65], is relatively poor. When CRC cells metastasize, they go through the process of epithelial to mesenchymal transition (EMT). This process has been described as promoting cancer stemcellness underlying tumor progression and intravasation allowing dissemination to distant organs, particularly in the liver and the lungs [66]. It is widely accepted that metastasis specifically to these organs is explained by their anatomical location, being the first and second draining sites of the colon respectively [67,68]. Additionally, according to the “seed and soil” hypothesis, the growth and metastatic behavior of CRC is dependent on tumor cells with stemness features endowed with self-renewal and migration properties (the “seed”) together with the microenvironment, on both local and systemic levels (the “soil”) [69,70,71,72]. The intrinsic immunosuppressive microenvironment of the liver could therefore facilitate metastatic spread to this organ [73].

Detailed characterization of the immune microenvironment of CRC liver metastases has shown that a high TIL density at the IM is associated with improved clinical prognosis in patients treated with chemotherapy [74]. Analysis of specific cells, associated with Th_1_ immune response, has demonstrated that compared with the immune phenotype in primary tumors, liver metastases are overall more frequently associated with high immune infiltrate for CD3^+^ T cells and CD45RO^+^ memory T cells in the IM, and CD8^+^ CTLs in both IM and CT, while CD20^+^ B-cell and FoxP3^+^ T-reg densities were higher in the CT of the primary tumors [75]. Increasing evidence suggests that the immune phenotype of CRC liver metastases frequently deviates from the corresponding primary tumor [75,76,77]. In 107 metastatic colorectal patients, no significant correlation was found between immune cell density in the primary tumor and matched metastatic lesion [75]. Furthermore, in 3 out of 16 patients with metastasized CRC, CD3^+^, CD8^+^ and granzyme B^+^ T cell densities were high in the primary tumor, while densities of these cells were low in corresponding liver metastasis [76]. The reverse, low levels in primary tumor and high levels in metastasis, was observed in 4 out of 16 patients. This indicates a substantial discordant immune-specific prognostic classification (7/16) [76].

For CRC lung metastasis, the infiltration pattern of CD8^+^ CTLs, DC-LAMP^+^ and NKp46^+^ cells did correlate between the primary tumor and the relapsing metastasis, as analyzed by immunohistochemistry [78]. Reasons for the observed difference between correspondence between the immune TME of the primary tumor and immune TME in either liver or lung metastatic sites are unclear, but highlight that factors shaping the immune TME depend on the site of metastasis. Recently, prognostic values of the Immunoscore of primary tumors and an Immunoscore-like classification of distant metastases, including liver, lung, distant lymph node and ovaries, were compared in 196 advanced CRC patients [77]. In multivariate analysis, the immunoscore derived from the metastatic lesion remained an independent prognostic marker, while the Immunoscore of the primary tumor lost its significance [77]. These findings suggest that immune infiltration at the metastatic site has a high impact on clinical outcome of patients with advanced CRC, surpassing the impact of infiltration to the primary tumor, and should be evaluated separately.

Little is known about the relation between molecular immune profiles of primary CRC tumor and paired metastasis. The majority of studies comparing transcriptomics of matched primary and metastatic tissue have selectively focused on the tumor compartment. For example, a study comparing matched primary- and metastatic samples of 13 CRC patients focused on metastasis-specific gene expression by filtering out all transcripts that are differentially expressed in primary tumor versus healthy colon and metastasis versus healthy liver such as immune related-genes [79]. Similarly, analysis of tumor epithelial cells by laser capture microdissection [80] and strategies aiming to identify genes exhibiting patterns of deregulation in metastases across patients, did not delineate immune molecular profiles [81,82]. In light of recent advances of targeted (immuno)-therapies, it will be crucial to define the immune gene signatures in tumor metastases that enable selection of the most beneficial therapy.

## 3. Predictive Signatures

To date, tumor location and TNM stage have been major decisive factors guiding therapy for CRC. The treatment of colon cancer comprises surgical resection of the primary tumor and regional lymph nodes followed by adjuvant chemotherapy, consisting of oxaliplatin, fluorouracil and leucovorin, in stage III and high-risk stage II colon cancer patients [83]. Patients with rectal cancer are typically treated with neoadjuvant radiotherapy or chemoradiation treatment followed by surgical resection [84]. More recently, MSI status has been recognized as a predictive factor in early-stage CRC, as tumors that exhibit MSI do not benefit from adjuvant 5-fluorouracil (5-FU) treatment in contrast to MSS CRC [85,86,87,88]. Additionally, identification of *KRAS*, *NRAS* and *BRAF* mutations can serve as predictive markers for targeted anti-EGFR therapy in patients with metastatic CRC [89]. These developments enable adjustment of therapy according to specific tumor characteristics and thereby provide the first steps towards personalized treatment. As components of the TME, including lymphocytes and stromal cells, have a significant impact on CRC progression, it is not surprising that the TME also influences antineoplastic treatment efficacy. In this section, the predictive value of the intratumoral immune phenotype will be reviewed in the context of both conventional therapies and immunotherapies that are currently under investigation.

### 3.1. Immune Signatures Predictive for Conventional Therapy

Although the direct elimination of tumor cells has been considered the primary mechanism of action of chemotherapy and radiotherapy, innate and adaptive immune responses seem to have substantial impact on treatment efficacy [90]. It is clear that conventional adjuvant anti-tumor therapies induce immune responses, either contributing to treatment efficacy or paradoxically stimulating tumor progression. Cancer cell death induced by conventional antineoplastic therapies elicits immune responses by release of tumor antigens, ATP and high mobility group box 1 (HMGB1). Released HMGB1 can either stimulate immune-mediated tumor death or contrarily induce a tumor-supportive inflammatory response, depending on its oxidation status [91]. Oxidized HMGB1 acts as a ligand of TLR4 on DCs and upon binding activates processing and presentation of antigens. In breast cancer, TLR4 mutation was shown to associate with decreased relapse free survival after radiotherapy and chemotherapy, demonstrating the clinical relevance of immunoadjuvant effect of these treatment modalities [90]. In immunocompetent mouse models of colon cancer, release of HMGB1 upon 5-FU chemotherapy or surgery has been associated with recruitment of MDSC to the tumor site, promoting inflammation, tumor growth and vascularization [92,93,94]. As most colon cancer patients receive combinations of different chemotherapeutic agents, their combined effect on the immune system and the induced changes in the TME should be considered in evaluating treatment outcome. Patients with advanced colorectal cancer displayed reduced levels of circulating MDSCs upon combined chemotherapy of folinic acid, 5-FU, and oxaliplatin (FOLFOX) treatment, reflecting a reduction of immunosuppression [95]. The reverse was observed in patients under folinic acid, 5FU, and CPT11 (FOLFIRI) treatment, revealing an increase in MDSC levels and increased immune suppression [95]. Radiotherapy also displays interactions with innate and adaptive immune responses by increased expression of MHC-I and MHC-II molecules, CD80, stress ligands and death receptors on tumor cell surfaces and release of immune-activating chemokines, cytokines, exosomes and danger signals hereby recruiting DCs to the tumor site [96,97].

To optimize therapy allocation, including the exploration of effective combinations of conventional therapies and immunotherapies, it is very important to be able to predict immune responses upon treatment in individual patients. A recent study has shown that stage II/II CRC patients with high *PD-L1* gene expression in their TME, have a poorer relapse free survival following standard 5-FU based adjuvant chemotherapy compared to the untreated setting, while patients with low *PD-L1* displayed a significant benefit from adjuvant treatment [98]. This suggests that *PD-L1* expression could be a negative predictive marker for adjuvant chemotherapy. As high *PD-L1* significantly correlated with increased immune cell infiltration and associated with MSI [98], these tumors mostly belong to CMS1 subtype and are potential candidates for immune checkpoint inhibitor-based immunotherapy as a primary treatment [50]. The biological rationale of the adverse effect of chemotherapy in *PD-L1* high tumors might be the loss of TILs caused by chemotherapeutics, hereby eliminating the pre-treatment equilibrium imposed by the immune infiltrate [99].

Similarly, accumulation of MDSCs and Th_17_ cells, both characteristics of a CMS4 immune contexture [18,45], were found predictive of a response of metastatic colorectal cancer to FOLFOX treatment in combination with the anti-*VEGF* antibody bevacizumab [100]. These immune parameters might simply serve as biomarkers to identify CMS4 tumors, which are also characterized by increased angiogenesis, explaining its response to the antiangiogenic agent bevacizumab. Additionally, FOLFOX-bevacizumab treatment has shown to decrease granulocytic MDSCs which in turn is associated with improved survival [100], suggesting an immunoadjuvant effect which might be particularly relevant for CMS4 tumors.

Treatment response to neoadjuvant chemo- and radiotherapy of rectal tumors seems improved by a locally activated Th_1_-type immune TME. Patients with high pre-treatment densities of intratumoral CD3^+^ and CD8^+^ TILs were shown to have an improved disease free and overall survival compared with patients with low pre-treatment CD3^+^ and CD8^+^ TIL levels [101]. Post-treatment samples displayed higher levels of TILs, suggesting that chemoradiation therapy can enhance pre-existing local immune responses [101]. Along these lines, an analysis of differentially expressed genes in pre-treatment biopsies identified six genes that were over-expressed in responders to neoadjuvant chemoradiotherapy versus non-responders: *CXCL11*, *HLA-DRA* and *MMP12* and ICR genes *CXCL9*, *CXCL10* and *IDO1* [102]. This supports the idea that an intratumoral Th_1_-type immune phenotype before chemoradiotherapy promotes the efficacy of this treatment modality. As the number of TILs are upregulated in the majority of rectal cancer patients receiving radiotherapy [101], application of immunotherapeutic strategies to further stimulate the anti-tumoral immune responses might increase the number of patients that respond to treatment. The combination of radiotherapy with immune checkpoint inhibition is currently being investigated in several ongoing clinical trials in both rectal cancer and other tumor types, including unresected pancreatic cancer, non-small cell lung carcinoma, advanced cervical cancer and metastatic melanoma [103]. Although a poor immunogenic TME is associated with no response to radiotherapy, it might not be applied as a predictive marker to exclude patients from this treatment, since the separation of biomarkers levels between responders and non-responders is narrow [101,102]. Potentially, it could be used to decide to precede radiotherapy with immunotherapeutics as radio-sensitizer.

### 3.2. Immune Signatures Predictive for Immunotherapy

While conventional therapies can elicit immune responses that contribute to their efficacy, immunotherapies are specifically aimed to induce anti-tumoral immune responses. Different strategies are being explored across multiple cancer types, including active approaches that enhance the host intrinsic anti-tumor immune response by cytokine treatment, immune checkpoint inhibition and vaccination. Other, passive approaches employ effector molecules or -cells developed outside the patients’ body, including adoptive T cell transfer and monoclonal antibodies targeting tumor-associated antigens [104]. So far, immune checkpoint inhibition has proven to be a powerful new therapeutic choice in melanoma, lung cancer, urothelial carcinoma, myeloma and renal cell carcinoma [105,106,107,108,109]. For CRC, clinical trials are still in early phases, since this cancer type was initially considered immunologically silent. It is now clear that CRC can indeed be targeted by immunotherapy. In particular, MSI tumors have proven to be responsive to anti-PD-1/PD-L1 therapy [110,111]. These tumors may specifically be targetable by immune checkpoint inhibitors because they typically exhibit an active immune TME, characterized by upregulation of ICR genes as well as immune checkpoint molecules such as PD-1, PD-L1, CTLA-4 and IDO [112]. As most CMS1 tumors display these attributes [18], these tumors represent optimal candidates for immune checkpoint inhibition (Table 1). Recently, anti-PD-1 treatment has been FDA-approved for tumors with MSI and defective DNA repair mechanisms. Additionally, combination of checkpoint inhibitors with peptide-based vaccinations targeting tumor associated neoantigens could further enhance anti-tumor immune responses [113]. While previous testing of peptide vaccine monotherapies has only resulted in modest anti-tumor responses, immune checkpoint antibodies potentiate the induced immune responses [114].

A different strategy to re-engage the immune system will be required for CMS4 tumors, as both immunosuppressive mechanisms and pre-existent immune cells are substantially different in these tumors (Figure 2, Table 1). As key aspects shaping the TME of these tumors include TGFβ signaling and an angiogenic microenvironment, targeting these attributes will be essential to transition to a “CMS1-like” immune TME. In mouse models of melanoma, silencing of either VEGF or TGFβ early in tumor formation completely changed the suppressed immune contexture to an effector-oriented one and restored sensitivity to immune checkpoint inhibitors, supporting that these are molecular drivers of the immune phenotype [127]. In a mouse model of mesenchymal CRC, a synergic effect was observed when targeting the TGFβ and PD-1 pathway [119]. At this moment, multiple TGFβ targeted therapies are in clinical trials for CRC, mainly driven by the marked pro-metastatic effect of TGFβ signaling [120]. The next step would be to test its combination with immune checkpoint inhibition in clinical trials. As mentioned, anti-angiogenic treatment by monoclonal antibodies directed against VEGF induce an adjuvant immune effect when combined with conventional chemotherapy [100]. This effect on the immune system can be further enhanced by combination with immune therapeutic approaches [122]. Several clinical trials are investigating the safety and efficacy of combining bevacizumab (anti-VEGF) with atezolizumab (anti-PDL-1) in CRC at this moment (NCT02873195, NCT02291289, NCT02876224, NCT01633970) [103]. Alternatively, strategies that specifically target the expansion, recruitment or immunosuppressive functions of cell populations including T-regs and MDSCs have been proposed [126]. In renal cell carcinoma, the tyrosine kinase inhibitor sunitinib, was shown to prevent MDSC accumulation resulting in restoration of a Th_1_-type immune infiltrate accompanied with a reduction in T-regs [125]. The effect of T-reg and MDSC inhibition in CRC patients, and its potential synergic effect with other immunotherapeutics, needs further investigation.

CMS2 and -3 tumors are typically poorly immunogenic and lack immune cell infiltration. To convert these “cold” tumors to “hot” tumors that are targetable by immunotherapeutic approaches, mechanisms responsible for the absence of intratumoral immune cells should be identified and reverted (Table 1). One of the mechanisms that confers CRC tumors less immunogenic is downregulation of MHC class I, resulting in reduced presentation of tumor-associated antigens [59]. Cobimetinib, a mitogen-activated protein kinase (MAPK) kinase (MEK/MAP2K) inhibitor, has been shown to promote MHC I expression, resulting in an accumulation of CD8^+^ CTLs in the TME [116]. MAPK signaling inhibition by cobimetinib is specifically suggested for *KRAS* mutated CRCs [117], which typically are CMS3 [50]. A recent clinical trial testing the combination of cobimetinib with anti-PDL-1 therapy in 22 metastasized CRC patients with mutated *KRAS* and one with wildtype *KRAS*, resulted in four partial responders [118]. Interestingly, three of the responders had intact mismatch repair, suggesting that combination treatment with immunotherapy can indeed be applied beyond patients with MSI CRC. Another attribute of poorly infiltrated CMS2 and CMS3 tumors is reduced expression of T cell attracting chemokines [18]. In a preclinical screen of different FDA-approved anti-cancer drugs, histone deacetylase (HDAC) inhibitors were found to increase expression of multiple T cell chemokines in cancer cells, macrophages and T cells, resulting in enhanced T cell infiltration and PD-1 sensitivity [121]. A phase I/II trial is currently ongoing to test the safety and efficacy of combining romidepsin, a HDAC inhibitor, with anti-PD-1 therapy in advanced colorectal cancer (NCT02512172).

A different strategy to stimulate immune cell infiltration to the tumor site is the combination of conventional chemotherapeutic strategies known to elicit immune responses [95] with immune checkpoint inhibition. A prove of concept was provided in a mouse model of lung adenocarcinoma that lacked T cell infiltration and was not responsive to checkpoint inhibition [123]. Treatment with oxaliplatin combined with cyclophosphamide was able to turn the tumor immune responsive and sensitive to checkpoint inhibition therapy [123]. Clinical trials testing the combination of anti-PDL-1/PD-1 treatment with radiotherapy or modified FOLFOX are underway (NCT02437071, NCT02375672). Similarly, the anti-EGFR monoclonal antibody cetuximab, mainly applicable to CMS2 tumors that are characterized by EGFR activation without mutations in downstream pathways (e.g., KRAS mutations), has a potential synergistic effect with immune checkpoint inhibitors [115]. Additionally, more passive immunotherapeutic treatments relying less on the endogenous immune system including cancer vaccines with primed DCs and adoptive transfer T cells therapies are under development [124]. These approaches may specifically increase benefit from immunotherapy to CMS2 and CMS3 CRC.

## 4. Conclusions

The recognition of the impact of the immune system on the progression of CRC has led to the identification and detailed characterization of tumor immune phenotypes. The type, location and density of TILs in combination with their functional molecular orientation, together defined as the immune contexture, determines the effect on patient prognosis. CMSs are associated with specific immune infiltration profiles corresponding with characteristic mechanisms of immune escape. At least three different immune phenotypes have been identified: (1) highly immune-infiltrated tumors with a favorable, reactive, Th_1_-type functional molecular orientation, (2) highly immune-infiltrated tumors with an unfavorable, inflamed molecular orientation and (3) poorly immunogenic tumors with no or minimal immune cell infiltration. Factors that shape the immune contexture, derived from tumoral cells as well as stromal cells in the TME, are now being elucidated. These insights are highly relevant for the design and allocation of therapeutic approaches that rely on the anti-tumoral immune responses, including both conventional treatment options (chemo- and radiotherapy) and immunotherapy. In theory, all CRC could be targeted by immunotherapy by shifting the tumor towards a “CMS1-like”, reactive immune phenotype through the application of the right combination of drugs. Undoubtedly, not all patients within a specific CMS subgroup will respond similarly to a specific treatment protocol. A crucial aspect in future clinical trial design will be to specify biomarkers beyond MSI status to allocate the right therapeutic strategy to each individual patient. Furthermore, in patients with metastasized CRC specifically, determination of the immune phenotype in the tumor metastasis is required since it has a dominant influence on clinical outcome. Foremost among the needed extra data is tumor gene expression data from consecutive samples collected before, during and after treatment, which will be of tremendous importance to further increase our understanding on how to manipulate the immune TME. This knowledge will bring us one step closer to be able direct the immune system toward tumor rejection in all patients.

## Figures and Tables

**Table 1:** Potential strategies for immunotherapy across consensus molecular subtypes (CMS) of colorectal cancer (CRC).

CMS1	CMS2	CMS3	CMS4
Immune checkpoint inhibition (anti-PD-1/PD-L1, anti-CTLA-4, anti-IDO) [110,111,112]	Combined EGF pathway inhibition and immune checkpoint inhibition [115]	Combined MEK-inhibitor and immune checkpoint inhibition [59,116,117,118]	Combined TGF pathway inhibition and immune checkpoint inhibition [119,120]
	Combined HDAC inhibitors and immune checkpoint inhibition [121]	Combined HDAC inhibitors and immune checkpoint inhibition [121]	Combined angiogenesis blockade and immune checkpoint inhibition [100,122]
Combined neoantigen-based peptide vaccination and immune checkpoint inhibition [113,114]	Immuno-chemotherapy [123]	Immuno-chemotherapy [123]	
	Passive immunotherapy (DCs vaccines, ACT) [124]	Passive immunotherapy (DCs vaccines, ACT) [124]	Anti-T-reg and/or anti-MDSCs treatment [125,126]

Proposed strategies to apply immunotherapeutic approaches across all types of CRC supported by preclinical or clinical evidence. PD-(L)1: programmed death (ligand) 1, CTLA-4: cytotoxic T-lymphocyte-associated antigen 4, IDO: indoleamine 2,3-dioxygenase, EGFR: epidermal growth factor receptor, HDAC: histone deacetylase, DC: dendritic cell, ACT: adoptive cell transfer, MEK: mitogen-activated protein kinase (MAPK) kinase, T-reg: T-regulatory cells, MDSCs: myeloid derived suppressor cells, TGF: transforming growth factor.

## Abbreviations

5-FU	5-fluorouracil
CAF	Cancer associated fibroblast
CMS	Consensus molecular subtype
CRC	Colorectal cancer
CT	Core of the tumor
CTL	Cytotoxic T lymphocyte
DC	Dendritic cell
DC-LAMP^+^	Dendritic cell lysosome-associated membrane glycoprotein positive
EMT	Epithelial to mesenchymal transition
HLA	Human leukocyte antigen
ICR	Immunologic constant of rejection
IM	Invasive margin
MDSC	Myeloid derived suppressor cell
MEK	Mitogen-activated protein kinase (mapk) kinase
MHC	Major histocompatibility complex
MSI	Microsatellite instability
MSS	Microsatellite stable
NK	Natural killer cell
Th	T-helper cell
TIL	Tumor infiltrating lymphocyte
TLS	Tertiary lymphoid structure
TME	Tumor microenvironment
TNM	Tumor node metastasis
T-reg	Regulatory T cell
